# Determinants of *Schistosoma mansoni* transmission in hotspots at the late stage of elimination in Egypt

**DOI:** 10.1186/s40249-022-01026-3

**Published:** 2022-09-23

**Authors:** Ramy Mohamed Ghazy, Walid Ismail Ellakany, Mai M. Badr, Nehad E. M. Taktak, Heba Elhadad, Sarah M. Abdo, Ayat Hagag, Abdel Rahman Hussein, Mohamed Mostafa Tahoun

**Affiliations:** 1 Tropical Health Department, High Institute of Public Health, Alexandria University, Alexandria, Egypt; 2 Department of Tropical Medicine, Faculty of Medicine, Alexandria University, Alexandria, Egypt; 3 Department of Environmental Health, High Institute of Public Health Alexandria University, Alexandria, Egypt; 4 Parasitology Department, Medical Research Institute, Alexandria university, Alexandria, Egypt; 5 Department of Medical Parasitology, Faculty of Medicine, Kafrelsheikh University, Kafrelsheikh, 35516 Egypt; 6 Ministry of Health and Population, Alexandria, Egypt; 7 Department of Epidemiology, High Institute of Public Health Alexandria University, Alexandria, Egypt

**Keywords:** *Schistosoma mansoni*, Praziquantel, *Biomphalaria alexandrina*, Community engagement, Transmission cycle, Neglected tropical diseases

## Abstract

**Background:**

In certain settings, the prevalence and severity of schistosoma infection do not lessen despite repeated rounds of preventative chemotherapy; these areas are known as hotspots. This study aimed to investigate the role of human practices, besides environmental and malacological factors, in the maintenance of the *Schistosoma mansoni* infection transmission chain in hotspot areas in Egypt.

**Methods:**

This cross-sectional study was conducted between July and November 2019 in Kafr El-Sheikh Governorate, Egypt. A pre-designed structured interviewing questionnaire was used to collect humanitarian data. Stool samples were collected from children aged 6–15 years on three successive days and examined using the Kato-Katz technique. Simultaneously, water and snail samples were taken from watercourses surrounding houses. Snails were identified based on their shell morphology and structure and tested for cercaria shedding. Water samples were analyzed for their physicochemical and biological characteristics.

**Results:**

A total of 2259 fecal samples (1113 in summer and 1146 in fall) were collected from 861 children. About 46.9% of the participants were males, and 31.8% were aged 6–10 years. The prevalence of *S. mansoni* infection was higher during the summer than during the fall (19.1% vs 7.2%, respectively, *P* < 0.01). The intensity of infection (light, moderate, and heavy) during summer versus fall was (93.55 vs 89.38%, 6.45 vs 8.85%, and 0.00% vs 1.77%), respectively (*P* < 0.05). A higher prevalence of human infection was observed among males than females [*OR* = 1.63, 95% confidence interval (*CI*):1.10–2.40, *P* = 0.015], children aged 11–15 years than among their counterparts aged 6–10 years (*OR* = 2.96, 95% *CI*: 1.72–5.06, *P* < 0.001), and mothers with a low level of education (*OR* = 3.33, 95% *CI*: 1.70–6.52, *P* < 0.001). The main identified risk factors were contacting the main body of water-canal for washing clothes (*OR* = 1.81, 95% *CI*: 1.12–2.49, *P* = 0.015), land irrigation (*OR* = 2.56, 95% *CI*: 1.32–4.96, *P* = 0.004), water collection (*OR* = 2.94, 95% *CI*: 1.82–4.73, *P* < 0.001), bathing (*OR* = 2.34, 95% *CI*: 1.21–4.31, *P* = 0.009), and garbage disposal (*OR* = 2.38, 95% *CI*:1.38–4.12, *P* < 0.001). The count of *Biomphalaria alexandrina* was distinct between seasons (*P* < 0.01) in consistent with statistically significant differences in water temperature, salinity, turbidity, the total concentration of coliforms, depth, velocity, and water level (*P* < 0.01). The presence of grasses and duckweeds was significantly associated with snail infection (*P* = 0.00 l). Significant effects of water depth, pH, temperature, and total dissolved solids on snail count were also observed (*P* < 0.05).

**Conclusions:**

The persistence of the infection is due to adoption of risky behaviors and environmental factors that enhance snail survival and infection. Schistosomiasis elimination in hotspots requires an integrated control approach that combines preventive chemotherapy with other complementary measures.

## Background

Schistosomiasis is a neglected tropical disease (NTD) caused by a blood fluke of the genus *Schistosoma*; however, it is regarded as one of the most important parasitic diseases [1]. In 2019, infection persisted in 78 countries, with 240 million cases reported necessitating chemoprophylaxis for approximately 236.6 million people [1, 2]. The Middle Eastern and North African (MENA) region is an endemic area for schistosomiasis. In 2010, approximately 7.2 million Egyptians were infected with schistosomiasis [3]. Schistosomiasis control programs rely heavily on mass drug administration (MDA) because of their high sustainability and effectiveness [4]. However, schistosomiasis transmission is still maintained by the release of eggs via urination *(Schistosoma haematobium*) or defecation (*S. mansoni*) into freshwater inhabited by snails that serve as intermediate hosts; humans become infected by coming into contact with these water sources containing cercariae [1, 2].

Sustainable control of the disease necessitates an integrated multi-component strategy that includes praziquantel (PZQ) chemotherapy, snail control, health education, improved water supply, sanitation, and management of infected watercourses [5]. Undoubtedly, behavioral change is one of the core components of any disease control program and schistosomiasis control in particular. Identifying the underlying risky behaviors causing persistent transmission, despite repeated MDA, is crucial for ensuring the effectiveness of disrupting the continued parasite transmission in endemic communities [6, 7]. Furthermore, attention should be directed to non-human factors such as snail population growth and environmental parameters such as pH, salinity, temperature, rain, light, water current speed, vegetation, turbidity, and desiccation fluctuations that influence the persistence of schistosomiasis infection [8]. This has led to calls for the integration of multiple strategies, including providing safe water supply, sanitary waste disposal, and personal hygiene, with chemoprophylaxis as more effective socio-ecological measures for achieving the sustainable control of schistosomiasis, snail control, and other water-related infectious diseases [9–11]. The World Health Organization (WHO) Expert Committee on Epidemiology and Control of Schistosomiasis highlighted this fact early in 1978, stating that "creating long-term effective control programs for schistosomiasis requires a comprehensive understanding of environmental, demographic, social, human behavioral, and economic factors in the disease." [12].

Therefore, addressing the epidemiological aspects of schistosomiasis transmission and control as well as looking into the distribution and population dynamics of intermediate hosts are necessary for the implementation of disease control programs. This can be achieved through conducting epidemiological studies in endemic areas, considering that each site has its own unique biological, ecological, social, and economic characteristics [5, 13].

Intestinal schistosomiasis is an ancient endemic disease in Egypt targeted for elimination [14]. In 1985, MDA with PZQ administration strategy was implemented by the Egyptian Ministry of Health and Population (MOHP) and the United States Agency for International Development. PZQ was administrated to all schistosomiasis cases free of charge. In 1990 there was a 38% decline in *S. mansoni* overall infection prevalence in Nile Delta governorates. The establishment of the National schistosomiasis Control Program in the Nile Delta in 1996 reduced S. mansoni prevalence from more than 30% to less than 10% in 2010 [15]. MDA with PZQ was administrated to all school-children 6–8 years old in 1997. The strategy was supplemented with health education and molluscicides. Currently, the Egyptian MOHP adopted a multi-sectorial approach through the integration of mass treatment, health education, social mobilization, environmental sanitation, and snail control [14, 15].

Later studies reported different *S. mansoni* hotspots with a 15.2%, 26.6%, and 30.9% infection prevalence in villages near Alexandria, at the north of the Nile delta and in Kafr-El-Sheikh, respectively [16, 17]. The presence of these hotspots indicated that the project was incapable of breaking the cycle of transmission even when repeated on a regular basis [18]. The existence of hotspots may be attributed to high levels of transmission due to increased juvenile infection that requests modification in treatment strategy as PZQ is ineffective in premature infection [15], reduced susceptibility to PZQ [19], and unavailability of latrines and sanitary disposal [14, 18]. One of the main current challenges to fighting schistosomiasis in hot spot areas is to extend MDA administration to cover100% of geographical areas, provide MDA to more than 75% of school-aged children in endemic areas, and reach the adult high-risk population as irrigation workers, fishermen, and females. This population may be missed throughout deworming rounds [15, 20].

In 2016, unpublished data from a project titled “Evaluation of PCR Assay for Detection of *Schistosoma mansoni* DNA in Human Stool Samples,” funded by the WHO Eastern Mediterranean Regional Office (WHO/EMRO), revealed a prevalence of 30% among children aged 6 to 15 years, despite the MOHP) effective MDA strategy [21]. Based on this finding, we aimed to address responses to several questions regarding the effect of human risky behaviors besides environmental, biosocial, and economic factors that may have played a crucial role in maintaining the transmission cycle in hotspots in Kafr El-Sheikh, Egypt.

## Methods

### Study design

**Fig. 1:**
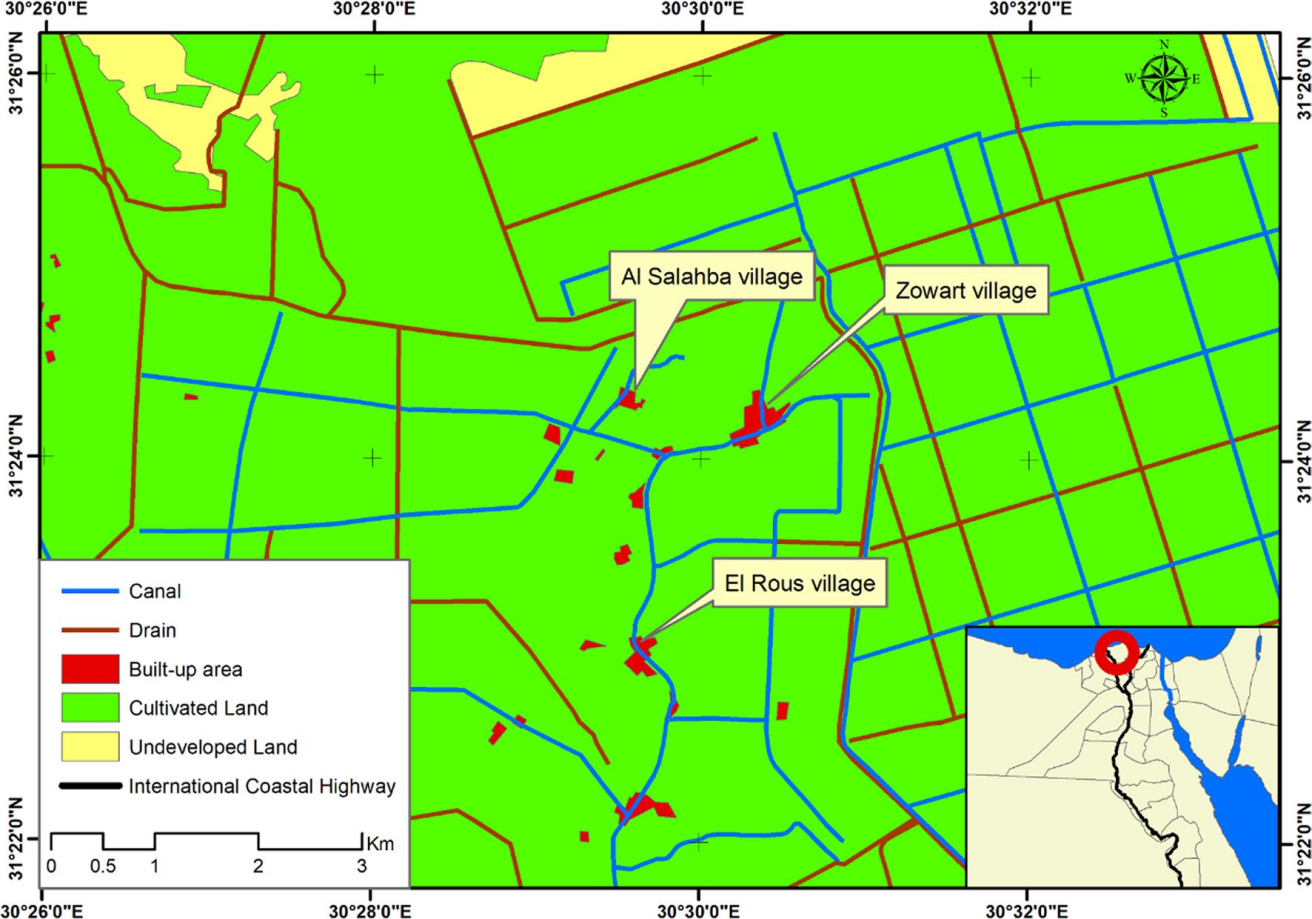
Map of Kafr El-Sheikh Governorate showing the studied villages

A cross-sectional study was conducted in three Kafr El-Sheikh villages, El-Roos (Village I), El-Salahba (Village II), and El-Zowarat (Village III) (Fig. 1), where *S. mansoni* infection is still circulating, based on national data where persistent transmission of *S. mansoni* infection is still reported. The study was conducted between July and November 2019 recruiting locally resident children aged 6–15 years old, free of chronic disease, and with neither history of recent treatment with PZQ within the preceding 3 months nor being physically handicapped.

### Study site

Kafr El-Sheikh is one of the Egyptian Governorates located in the north along the west branch of the Nile in the Delta region, at 31°18 North and 30°55’ East. It has a total land area of 34,671 square kilometers.

### Sample size

As the reported prevalence of *S. mansoni* among children in Kafr El-Sheikh is 30% [21], with 5% precision, and a 95% confidence interval (*CI*), a minimum sample size of 323 children was recruited (Epi-Info 7.2). In total, 861 children were included in two-time points (432 during summer & 429 during fall) and each child was surveyed once in either season.

During the summer, when children were not in school, quota sampling was used to select children in proportion to their population density. Recruits were gathered from gathering places such as markets. During the fall season, a multistage stratified random sampling technique was implemented. Three schools were included to represent the selected villages. Each school had nine grades (six primary grades and three preparatory grades) divided into classes. Half of the classes were selected at random, and students were proportionately allocated according to the number of students in each grade. A simple random sampling technique was used to recruit students from the classes.

### Data collection

Based on the literature review, pilot study, and community engagement and interview, a pre-designed structured interviewing questionnaire was used to collect data from the children, including: Humanitarian factors (age, sex, parental education, socioeconomic status), housing conditions (residence, house proximity to water canal, water shutoff), risky behaviors (contact with water canal, site of contact), cause of contact (washing clothes, irrigation, water collection, bathing, garbage disposal), and health care services utlization were analyzed in the section of human study (previous PZQ treatment, visiting the local health unit). The socioeconomic status was evaluated based on Fahmy and Nofal’s scoring system [22]. The survey was uploaded to the Survey-Monkey server, and data collectors were well versed in online data collection procedures. The responses were reviewed in real-time review, and data was verified for completeness and accuracy by senior field supervisors (RMG). Environmental factors included water depth, velocity, level, pH, temperature, turbidity, TDS, TC, *Escherichia coli* (*E. coli*), and vegetation. The malacological survey included snail collection, species identification, and prevalence of infection with *S.mansoni* .

#### Laboratory methods

All laboratory procedures were conducted in the Central Laboratory of the High Institute of Public Health (HIPH), Alexandria University, Egypt.

#### Parasitological methods

Children’s fresh stool samples were collected on three consecutive days. A total of 2259 fecal samples were collected from 861 children, 1113 in the summer; [Village I (328), Village II (523) and Village III (262)] and 1146 in the fall [Village I (397), Village II (192) and Village III (557)]. Using the Kato-Katz technique, stool samples were examined; two slides were prepared for each sample using 41.7 mg plastic templates [23], for a total of six slides per child. The number of eggs per slide and the infection intensity (the geometric mean of egg excretion by infected individuals) were calculated [24]. The quality of the used reagents and instruments was checked. Two specialists in parasitology double-checked the positive results and a random sample of the negative results.

#### Environmental and malacological methods

Water and snail samples were collected twice during each season from the watercourses surrounding the three villages: Village I (2 watercourses), Village II (4 watercourses), and Village III (two watercourses), according to standard sampling procedures [25, 26].

#### Environmental sampling

The samples were collected at the beginning, middle, and end of the selected water course. The station length varied based on the length of the watercourse that ranged between 150 and 200 m. The following environmental indices were measured: temperature (using a standard thermometer at midday), pH (using a portable pH meter [A221] from Thermo Fisher Scientific, city, USA), salinity and total dissolved solids (TDS) by using a multimeter (SympHony Inc., city, USA), water velocity (by placing a very light floating object at the head of the canal and allowing it to move with the water current), turbidity (by HI88703-01 benchtop turbidity meter, Clarkson Laboratory and Supply Inc., city, USA), total coliform (TC) and E. coli by multiple tube technique, water depth (by using a long rope with known length fixed to stone) and vegetation distribution as a percentage and type.

#### Malacological sampling

Each watercourse was sampled five times along its length. Snail sampling was done by experienced field collectors using long-handled scoops and forceps for 15 min at each location in accordance with the standard procedures [25]. The collected snail specimens were transported to the laboratory in perforated plastic boxes. Shell morphology and structure were used to identify snails using standard identification keys [27]. After identification, snails that could serve as intermediate host snails (*Biomphlaria alexandrina*) were examined for cercaria shedding. The collected *cercariae* were subsequently morphologically identified using identification keys [27–30].

#### Data management and analysis

Data was collected using an online server, then it was extracted into Excel worksheets. Frequency distributions and cross-tabulation were utilized for range checking to ensure that all questions had valid codes. Statistical Packages for the Social Sciences for Windows (version 25 Armonk, IBM Corp, NY, United States of America) was used to analyze the data. In order to describe qualitative data, percentages and bar charts were employed. Using the Pearson chi-square *t*-test, the relationship between categorical variables was analyzed. If the chi-square assumption was violated, Fisher's exact or Monte Carlo corrections were utilized. Freedman’s test was used to examine the variance of monthly difference in infection status. Negative binomial regression was used to examine the relationships between the number of collected snails and independent variables [water salinity, turbidity, pH, temperature, TC, *E. coli*, water vegetation, depth, velocity, and level].

### Ethical considerations

This research was approved by the Ethics Committee of the HIPH and the Central Administration for Communicable and Endemic Diseases of the MOHP. The research team adhered to the International Guidelines for the Responsible Conduct of Research. All participants’ legal guardians provided written consent after being informed of the study’s purpose, procedures, consequences, and alternatives. Both confidentiality and anonymity were ensured. At the end of the study, the research team conducted three health education sessions for the residents of the three villages at their respective schools to deliver health messages regarding safe exposure to watercourses, how to avoid schistosomiasis, disease symptoms, and complications, and where to seek treatment.

## Results

### Prevalence and intensity of *S. mansoni* infection

**Fig. 2:**
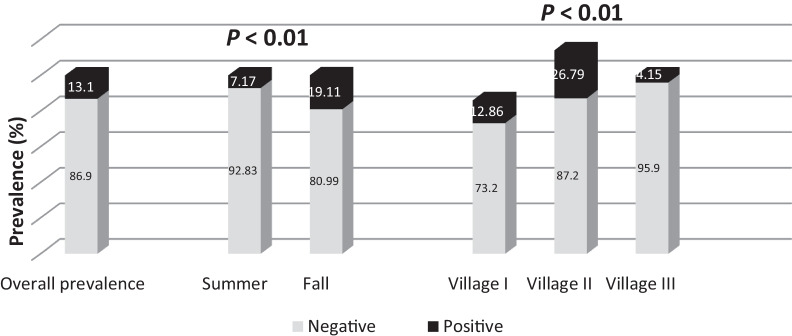
Prevalence of *Schistosoma mansoni* among the screened children (Kafr El-Sheikh governorate, 2019)

A total of 861 children aged 6 –15 years were surveyed in this study; 46.9% were males, and 31.8% were aged 6–10 years. Figure 2 shows the overall prevalence of *S. mansoni* infection; it was (13.1%, 113/861) among screened children in the two seasons. The prevalence of infection was significantly higher in the fall (19.11%; 82/429) than in the summer (7.17%; 31/432) (*χ*^2^ = 26.88, *P* < 0.01). There was a highly statistically significant difference in the prevalence of infection between the villages studied (*χ*^2^ = 44.5, *P* < 0.01); the highest prevalence in the two seasons combined was in Village II (26.79%), followed by Village I (12.86%), and then Village III (4.15%).

**Fig. 3:**
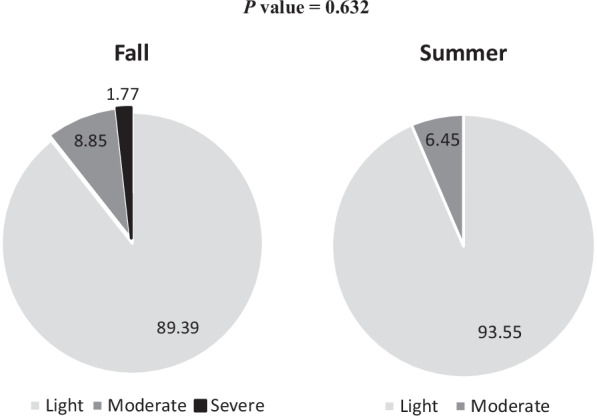
Intensity of schistosomiasis among the screened children in wet and dry seasons (Kafr El-Sheikh governorate, 2019)

As depicted in Fig. 3, light infection accounted for 93.55 and 89.39% of positive cases in the summer and in the fall, respectively. Moderate intensity was nearly identical in both seasons; 6.45% in the summer and 8.85% in the fall. However, only two cases (1.8%) were severely infected during the fall season.

### Determinants of *S. mansoni* infection

Table 1 shows that males and females accounted for 46.92% and 53.08%, respectively. The prevalence of infection was significantly higher among males (*OR* = 1.63, 95% *CI*: 1.10–2.43, *P* = 0.015), older children (*OR* = 2.92, 95% *CI*: 1.71–5.00, *P* < 0.001), children of illiterate mothers (*OR* = 3.33, 95% *CI*: 1.70–6.52, *P* < 0.001), residence (*OR* = 3.40, 95% *CI*: 1.71–6.79, *P* < 0.001) for Village I and (*OR* = 8.45, 95% *CI*: 4.12–17.35, *P* < 0.001) for Village II and children who reported frequent water unavailability (*OR* = 2.75, 95% *CI*: 1.72–4.14, *P* < 0.001). In contrast, there was no association between paternal education and the risk of infection (*OR* = 1.47, 95% *CI*: 0.92–2.34, *P* = 0.104). The prevalence of *S. mansoni* was higher in the low socioeconomic class than in the medium socioeconomic class (10.0% vs 13.98%), yet this difference was not statistically significant (*OR* = 1.44, 95% *CI*: 0.81–2.56, *P* = 0.212). Living close to watercourses was not significantly associated with acquiring infection (*OR* = 1.92, 95% *CI*: 0.82–4.54, *P* = 0.127).

**Table 1:** Socio-demographic and housing factors associated with *Schistosoma mansoni* infection (Kafr El-Sheikh, 2019)

Variable (*n* = 861)			Total *n* (%)	Negative *n* (%)	Positive *n* (%)	*OR* (95% *CI*)	*P*
Gender		Male	404 (46.9)	339 (83.9)	65 (16.1)	1.63 (1.10–2.43)	0.015*
		Female®	457 (53.1)	409 (89.5)	48 (10.5)	1.00	
Age		6–10 years	274 (31.8)	254 (93.8)	17 (6.2)	2.92 (1.71–5.00)	< 0.001**
		11–15 years®	587 (68.2)	491 (83.6)	96 (16.4)	1.00	
Mother level of education	Lower (*n* = 665)	Illiterate/Read and write	659 (76.5)	559 (84.8)	100 (15.2)	3.01 (1.70–6.52)	< 0.001**
		Literacy certificate	6 (0.7)	5 (83.3)	1 (16.7)		
	Higher® (*n* = 196)	Primary school	11 (1.6)	11 (100.0)	0 (0.0)	1.00	
		Preparatory school	34 (3.9)	33 (97.1)	1 (2.9)		
		Secondary school	122 (14.2)	113 (91.1)	9 (8.9)		
		University	29 (3.4)	28 (96.6)	1(3.3)		
Father level of education (*n* = 853)^#^	Lower (*n* = 597)	Illiterate/Read and write	580 (68.0)	497 (85.7)	83 (14.3)	1.47 (0.92–2.34)	0.104
		Literacy certificate	17 (2.0)	15 (88.2)	2 (11.5)		
	Higher® (*n* = 256)	Primary school	55 (6.4)	48 (87.3)	7 (12.7)	1.00	
		Preparatory school	44 (5.2)	38 (86.4)	6 (13.6)		
		Secondary school	107 (12.5)	100 (93.5)	7 (6.5)		
		University	50 (5.9)	44 (88.0)	6 (2.0)		
Socio-economic class		Low	711(82.6)	613 (86.2)	98 (13.8)	1.44 (0.81–2.56)	0.212
		Medium®	150 (17.4)	135 (90.0)	15 (10.0)	1.00	
Residence area		Village I	452 (52.5)	394 (87.2)	58 (12.8)	3.40 (1.71–6.79)	< 0.001**
		Village II	168 (19.5)	123 (73.2)	45 (26.8)	8.45 (4.12–17.35)	< 0.001**
		Village III®	241 (28.0)	231 (95.9)	10 (4.1)	1.00	
House proximity to water canal		Less than 30 min	782 (90.8)	675 (86.3)	107 (13.7)	1.92 (0.82–4.54)	0.127
		More than 30 min®	79 (9.2)	73 (92.3)	6 (7.6)	1.00	
Water shut-outs		Yes	144 (16.7)	113 (78.5)	31 (21.5)	2.75 (1.72–4.14)	< 0.001**
		No®	717(83.3)	652(90.9)	65 (9.1)	1.00	

**®** Reference value; ^#^the difference in the total number in is due to death of father;*: statistical significance *P* < 0.05; **: high statistical significance *P* < 0.01

*OR* Odd ratio, *CI* Confidential interval

Table 2 depicts the behavioral risks associated with *S. mansoni* infection and the effect of previously administered PZQ on the prevalence of *S. mansoni*. Indeed, a significant association was observed between contact watercourses and infection acquisition; based on self-reporting; 15.9% of children who reported contact with watercourses contracted the infection compared to 10.5% of those who reported no contact (*OR* = 1.62, 95% *CI*: 1.09–2.42, *P* = 0.018). Of the 421 children who reported contact with watercourses, those who contacted the main body were more likely to be infected than those who contacted the banks (23.6%) compared to (10.5%) (*OR* = 2.64, 95% *CI*: 1.26–5.52, *P* = 0.008) or both (24.7%) (*OR* = 2.80, 95% *CI*: 1.56–5.06, *P* < 0.001). Infection was more prevalent among children who engaged in the following risky behaviors; contact with watercourses for washing clothes (*OR* = 1.81, 95% *CI*: 1.12–2.94, *P* = 0.015), water collection (*OR* = 2.94, 95% *CI*: 1.82–4.72), irrigation (*OR* = 2.59, 95% *CI*: 1.33–5.33.) and bathing (*OR* = 2.94, 95% *CI*: 1.82–4.73, *P* < 0.001). Previous doses of PZQ had no effect on *S. mansoni* infection (*OR* = 0.68, 95% *CI*: 0.43–1.01, *P* = 0.095). Children who disposed trash in the canal had a significantly higher infection rate (24.2%) than those who disposed trash in designated collection areas or garbage collection vehicles (11.94%), (*OR* = 2.38, 95% *CI*: 1.38–4.12, *P* < 0.001). Notably, only 12% of those with a health issue visited the local government’s health care unit whereas 34.3 were visiting private clinics. This variable was not associated with increased odds ratio of infection acquisition (*P* > 0.05).

**Table 2:** Behavioral risk factors associated with *Schistosoma mansoni* infection among the examined children (Kafr El-Sheikh, 2019)

	Varaible (*n* = 861)		Total *n* (%)	Negative *n* (%)	Positive *n* (%)	*OR* (95%* CI*)	*P*
Contact with canal water							
Yes			421 (48.9)	354 (84.1)	67 (15.9)	1.62 (1.09–2.42)	0.018*
No®			440 (51.1)	394 (89.5)	46 (9.5)	1.00	
Site of contact (*n* = 421)^#^							
Bank®			257 (61.0)	230 (89.5)	27 (10.5)	1.00	
Main body			55 (13.1)	42 (76.4)	13 (23.6)	2.64(1.26–5.52)	0.008*
Both			109 (25.9)	82 (75.2)	27 (24.7)	2.80(1.56–5.06)	< 0.001**
Main causes for canal water contact^$^	Washing clothes	Yes	132 (15.3)	106 (80.3)	26 (19.7)	1.81 (1.12–2.94)	0.015*
		No®	729 (84.7)	642 (88.1)	87 (11.9)	1.00	
	Irrigation	Yes	53 (6.2)	40 (75.5)	13 (24.5)	2.56 (1.32–4.96)	0.004*
		No®	808(93.8)	708(87.2)	100(12.4)	1.00	
	Water collection	Yes	112 (13.0)	82 (73.2)	30 (26.8)	2.94 (1.82–4.73)	< 0.001**
		No®	749(87.0)	666(88.9)	83(11.1)	1.00	
	Bathing	Yes	68 (7.9)	53 (77.9)	15 (22.1)	2.34 (1.21–4.13)	0.009**
		No®	793 (92.1)	695 (87.6)	98(12.4)	1.00	
Previous PZQ treatment		No	272 (31.6)	244 (79.7)	28(10.3)	0.68 (0.43–1.01)	0.095
		Yes ®	589 (68.4)	504 (85.6)	85 (14.4)	1.00	
Visited health care facilities		HCU®	103 (12.0)	93 (90.3)	10 (9.7)	1.00	
		Governmental hospital	462 (53.7)	406 (87.9)	56 (12.1)	1.28 (0.63–2.60)	0.491
		Private clinic	296 (34.3)	249(84.1)	47 (15.9)	1.76 (0.86–3.62)	0.12
Garbage disposal		Canal water	82 (9.5)	62 (75.6)	20 (24.4)	2.38 (1.38–4.12)	< 0.001**
		Garbage collection vehicles/areas®	779 (90.5)	686 (88.1)	93 (11.9)	1.00	

® is reference value; ^#^*n* = 421 as this is the total number of participants who contacted canal water; ^$^this is a multiple response question and were asked even the respondent replied “no” to contact with water canal question; *: statistical significance *P* < 0.05; **: high statistical significance *P* < 0.01

*OR* Odd ratio, *CI* Confidential interval, *PZQ* Praziquantel

### Correlation between human infection and snail infection

**Fig. 4:**
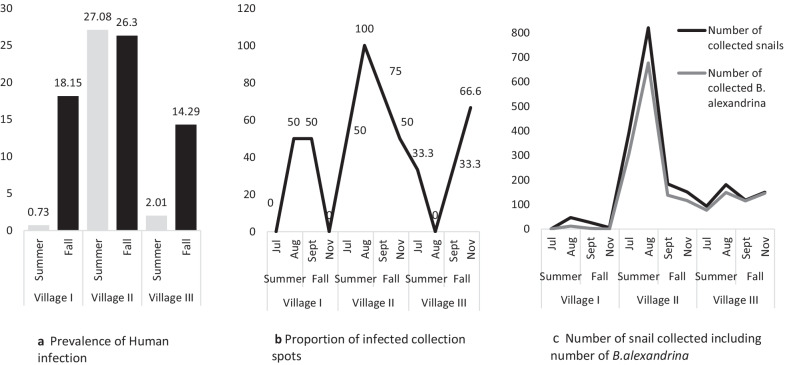
**a** Prevalence of human infection in different seasons (**b**), infected collection spots among the screened canals in each village at different (**c**), number of collected snails in different seasons including *Biomphalaria alexandrina* (Kafr El-Sheikh governorate, 2019). Malacological samples were collected from two canals for each month in village I, four canals for each month in village II, and three canals for each month in village III

The correlation between the prevalence of human infection and snail infection within each month is depicted in Fig. 4. In Village I, the prevalence of human infection increased from 0.7% in the summer to 18.2% in the fall, meanwhile, the prevalence of snail infection in different collection spots ranged from (0–50%) in both seasons. In Village II, the prevalence of *S. mansoni* infection was 27.0% during the summer, concurrent with 50%-100% *B. alexandrina* infection from the associated watercourses, and was nearly the same during the fall (26.3%), concurrent with 50% –75% *B. alexandrina* infection. In Village III, the prevalence of human infection ranged from 2.0% to 12.3% during summer and fall, respectively, while infected snails with cercaria in the included watercourses ranged from 0 to 33.3% and 33.3 to 66.6% during summer and fall, respectively.

### Malacological and water canal sampling findings

The month-specific malacological, physicochemical, and biological factors are presented in Table 3. Generally, among 45 spots studied in each month, the median absolute number of snails per study site was greatest in August [7(32.5)]; similarly, *B. alexandrina* was greatest in the same month [2(17.5)]. This difference was statistically significant, *P* = 0.005. Concerning water salinity, the highest concentration was recorded in November (363.10 mg/L), the lowest was in July (253.50 mg/L), and interestingly, it was nearly identical in August and September. Statistically, there was a statistically significant variation in monthly temperatures (*P* < 0.01). Between months, there were no statistically significant differences in the TDS concentration. Turbidity dropped from 13 Nephelometric turbidity unity (NTU) in July to around 9 NTU in both August and September, otherwise, its peak was recorded in November (16 NTU). This change was statistically significant (*P* = 0.003). For both total coliforms (TC) and Ecoli., which had their highest level in November (4.47 × 10^5^ and 3.99 × 10^4^, respectively) and the lowest in September (1.10 × 10^3^ and 1.69 × 10^2^, respectively), this fluctuation was also statistically significant (*P* < 0.001). The vegetation percentage was the same in July and September (80.0%), whereas in August and November it was (60.0% and 90.0%) respectively, however, this difference was not statistically significant (*P* = 0.08).

**Table 3:** Variation in the malacological and environmental factors of the screened watercourses across different months (Kafr El-Sheikh, 2019)

Parameter	Month median (IQR)				*Z*	*P*
	July	August	September	November		
Number of collected snails/ spot	2.00 (10.00)	7.00 (32.50)	0.00 (5.50)	0.00 (5.50)	12.74	0.005**
Number of *B. alexandrina*	0.00 (3.50)	2.00 (17.50)	0.00 (3.00)	0.00 (3.00)	12.94	0.005**
Salinity(mg/L)	253.50 (215.75)	352.90 (305.32)	351.07 (218.80)	363.10 (346.87)	12.39	0.006**
pH	6.81 (0.30)	6.87 (0.17)	6.90 (0.15)	6.85 (0.21)	2.87	0.412
Temperature (^°^C)	31 (0.00)	32 (0.00)	30 (0.00)	29 (0.00)	135	< 0.001**
TDS (mg/L)	360.00 (450.17)	463.67 (381.00)	462.00 (276.50)	448.00 (224.50)	3.36	0.339
Turbidity (NTU)	13.09 (18.89)	9.65 (26.76)	9.00 (13.75)	16.00 (27.74)	13.92	0.003**
TC (MPN/100 ml)	1.62 × 10^4^ (4.49 × 10^4^)	1.10 × 10^5^ (9.49 × 10^5^)	1.10 × 10^3^ (4.59 × 10^5^)	4.47 × 10^5^ (1.60 × 10^6^)	31.89	< 0.001**
*Escherichia coli* (MPN/100 ml)	2.20 × 10^3^ (1.78 × 10^3^)	4.66 × 10^3^ (1.25 × 10^5^)	1.69 × 10^2^ (1.09 × 10^5^)	3.99 × 10^4^ (8.45 × 10^4^)	31.41	< 0.001**
Vegetation (%)	80.00 (30.0)	60.00 (70.00)	80.00 (90.00)	90.00 (60.00)	6.66	0.084

*TC* Total coliforms, *TDS* Total dissolved solids, *IQR* Interquartile range; *: statistical significant *P* < 0.05; **: high statistical significance *P* < 0.01

Water depth reached more than 1 m (27.7%) in July before dropping to less than 0.5 m (35.7%) in August. Afterward, it rose to a range between 0.5 and 1 m in September (47.6%) and became > 1 m in November (29.8%). These differences in water depth were of a highly statistically significant difference (*χ*^2^ = 32.04, *P* < 0.001). Water velocity ranged from 0.5 to 1 m/sec in July (100%), followed by a peak reaching > 1 m in August (100%). Thereafter, it declined to less than 0.5 m in September (45%) and was finally still in November (29.6%). These monthly differences were also highly statistically significant (*χ*^2^ = 14.61, *P* < 0.001). Low water levels were observed in July and August (27.9 and 34.9% respectively). In September, 90% of the water levels were flooded (90%), whereas in November, the water level returned to normal (36.6%). The previously mentioned change in water level was of a high statistical significance (*χ*^2^ = 55.51, *P* < 0.001). Table 4

**Table 4:** Variation in water depth, velocity, and water level of the screened watercourses across different months (Kafr El-Sheikh, 2019)

Variable	Month *n* (%)				*χ*^2^	*P*
	July	August	September	November		
Depth (m)						
< 0.5 m	29.0 (25.9)	40.0 (35.7)	17.0 (15.2)	26.0 (23.2)	32.04	< 0.001**
0.5–1 m	3.0 (14.3)	3.0 (14.3)	10.0 (47.6)	5.0 (23.8)		
> 1 m	13.0 (27.7)	2.0 (4.3)	18.0 (38.3)	14.0 (29.8)		
Water velocity (m/s)						
> 1 m^3/^s	0.0 (0.0)	5.0(100.0)	0.0 (0.0)	0.0 (0.0)	14.61	0.002**
0.5–1 m^3/^s	3.0 (100.0)	0.0 (0.0)	0.0 (0.0)	0.0 (0.0)		
< 0.5 m^3/^s	4.0 (20.0)	7.0 (35.0)	9.0 (45.0)	0.0 (0.0)		
Still	38.0 (25.0)	33.0 (21.7)	36 (23.7)	45.0 (29.6)		
Water level						
Flooded	0.0 (0.0)	0.0 (0.0)	9.0 (90)	1.0 (10)	55.15	< 0.001**
Normal	9.0 (22.0)	0.0 (0.0)	17.0 (41.5)	15.0 (36.6)		
Low	36.0 (27.9)	45.0 (34.9)	19.0 (14.7)	29.0 (22.5)		

Statistical significance *P* < 0.05; **: high statistical significance *P* < 0.01

**Fig. 5:**
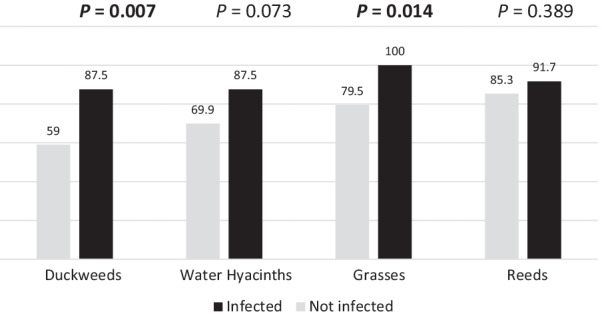
Types of vegetation associated with infection (Kafr El-Sheikh governorate, 2019)

Using negative binomial regression analysis, the main determinants of the number of *B. alexandrina* collected were water depth (β -1.8 and -0.89) for the depth of 0.5 –1 m and greater than 1 m respectively, water pH, and TDS (β = 2.61& -0.01) (Table 5). Both grasses and duckweeds had a significant association with snail infection (p = 0.009 and 0.006 respectively), indicating that plant type is a significant factor in the snail’s infection (Fig. 5).

**Table 5:** Predictors of snail population growth (Kafr El-Sheikh, 2019)

Total count of *Biomphalaria alexandrina*	β	*OR* (95% *CI*)	*P*
(Intercept)	− 15.37	0.00 (0.0001–0.002)	< 0.001**
Depth			
< 0.5 m®		1.00	
0.5–1 m	− 0.89	0.41(0.012–0.82)	0.01*
> 1 m	− 1.80	0.17 (0.001–0.31)	< 0.001**
pH	2.61	13.54 (3.36–54.54)	< 0.001**
TDS (mg/l)	− 0.01	0.99 (0.60–0.98)	< 0.001**
Temperature	− 0.53	0.59 (0.44–0.78)	< 0.001**

Reference value ® is < 0.5 m; Statistical significance *P* < 0.05; **: high statistical significance *P* < 0.01

*TDS* Total dissolved solids, *OR* Odd ratio, *CI* Confidential interval

## Discussion

The current strategy for schistosomiasis elimination is primarily based on preventive chemotherapy with periodic administration of the anti-schistosomal drug (PZQ) to school-aged children and other high-risk groups [30]. PZQ reduces morbidity and might has an impact on transmission, but rarely eliminates infection [31, 32].

This study aimed to identify the different ecological factors responsible for the sustained transmission of diseases in three hotspot areas of the Governorate of Kafr El-Sheikh. Seasonal variation and its influence on environmental factors affecting the prevalence of human and snail infection were the primary variables examined. In addition, sociodemographic and behavioral risk factors may play a significant role in maintaining the infection cycle.

### Seasonal variation and schistosomiasis prevalence in snails and humans

In this study, the overall prevalence of *S. mansoni* infection was (13.1%); 7.2% (31/432) in the summer and 19.2% (82/429) in the fall. The studied villages were considered to have low and moderate endemicity during summer and fall, respectively [24]. The current prevalence of schistosomiasis is relatively lower than that which was previously reported two years earlier in Arab El-Mahder village (30%), Kafr El-Sheikh. In Village II, the prevalence of infection was nearly identical during the summer and fall (26.3% and 27.1%, respectively), whereas it increased from 0.7% in the summer to 18.2% in the fall in Village I. This low prevalence during the summer can be attributed to chemotherapy campaigns conducted four months prior to the survey or to the difference in sampling technique between the two seasons. It is important to note that the number of *B. alexandrina* snails collected per location in the summer was greater than in the fall with an equal proportion of infected watercourses in each season. However, the prevalence of human infection was greater in the fall. This disparity between human and snail infection can be attributed to human activity; residents of Village I preferred to swim in the waterways of other villages where their farms were located. In addition, Village II’s watercourses were severely polluted and had a high water level, resulting in a low snail population despite the high prevalence of human infection. In addition to the fact that snail prevalence is not the only predictor of human infection, the point prevalence of *S. mansoni* infection should not be used to estimate the annual prevalence of infection.

### Intensity of infection

Indeed, the effectiveness of MDA programs for *S. mansoni* is mainly monitored by measuring changes in infection prevalence, drug treatment coverage, and the prevalence of heavy infection (≥ 400 epg) [1]. Regarding intensity of infection, the majority of children in this study 98.2% (111/113) had either light or moderate infection, and the prevalence of infection was either between 10%–50%. This finding could be due to the intensive MDA campaign implemented by the Egyptian MOHP.

### Sociodemographic factors

#### Age

In endemic areas, the infection is usually acquired during childhood [33]. The prevalence and intensity of infection rise with age and peaks at approximately 15 to 20 years. In older adults, the prevalence of infection does not change significantly, but intensity (parasite burden) decreases dramatically [34]. In this study, the mean age of infected children was significantly older than the uninfected children. Additionally, the age group of 11–15 years was more susceptible to infection than the age group of 6–10 years. Indeed, children aged 11–15 years can become more vulnerable for schistosomiasis when engaging in recreational activities such as swimming and playing in the water, when fetching water for household or agriculture activities. In the same line, many studies highlighted that different age groups had different susceptibility to infection. [35, 36]. On the other hand, a study conducted in Côte d’Ivoire found no difference in the prevalence of *S. mansoni* infection among the three investigated age groups [37].

#### Sex

The global distribution of schistosomiasis among both sexes is not fully addressed. However, most published surveys have found an equal prevalence of infection among men and women. However, the intensity of infection is more severe in females [38]. Nonetheless, in the current study, the prevalence of infection among girls was lower than that of boys, with no difference in intensity. We speculate that boys are more frequently exposed to water canals than girls. Moreover, the total surface area exposed to water varies due to a variety of water-related activities. Due to religious issues, customs, and traditions of the Egyptians, girls are prohibited from swimming in water canals while boys are permitted to do. Girls’ primary water activities include fetching water and washing clothes and dishes with their hands and legs only exposed. In line with this finding, another study conducted in Senegal found that males had higher infection rates [39]. Interestingly, the Côte d’Ivoire study found a similar prevalence among boys and girls; however, this may be due to the fact that significantly more boys (727 vs. 460) participated in the survey [37].

#### Education

In this study, maternal illiteracy was strongly associated with a higher prevalence of infection. In particular, our findings revealed that 74.7% of infected children had illiterate mothers. A study conducted in Santo Antonio de Jesus, Bahia State, Brazil, found that an increase in the education level of the household’s head was strongly associated with a reduction in the prevalence and intensity of infection in the household [40]. Nonetheless, the paternal education level had no significant effect on the prevalence of infection. Angora et al., [37] reached a similar conclusion; parental level of education was significantly associated with infection, but the maternal odds ratio was greater than three times the odds of paternal education. The higher risk associated with maternal illiteracy may be explained by the longer time mothers spend with their children and the profound influence they have on them.

#### Social class or status

Schistosomiasis is more prevalent in areas of poor socioeconomic conditions. Due to their low educational attainment, high unemployment rates, poor sanitary and housing conditions, and lack of access to health facilities, the inhabitants of these regions are at risk [12]. This study reported a higher prevalence in the low socioeconomic class (13.98%) than in the middle socioeconomic class (10%), but the difference was not statistically significant. The high social class effect could not be evaluated because most children were of low or intermediate social class. Extensive research has been conducted on the effects of poverty on the prevalence, incidence, and cost of schistosomiasis over time. Schistosomiasis  is a clear example of a disease caused by poverty [41].

In this study, watercourse proximity was not significantly associated with *S. mansoni* infection. In contrast, a detailed epidemiological study conducted in São Lourenço da Mata, Brazil, revealed that leisure water contact, particularly swimming, was the only type of water contact that was significantly associated with schistosomiasis among people between the ages of 10 and 25 and that better socioeconomic conditions were associated with a decrease in the frequency of water contact [42].

### Human water contact activities

Water contact is required in order to acquire schistosomiasis. However, 40.7% of infected children reported no contact with watercourses. This issue needs to be further discussed as if they did not encounter the water stream, what the supposed route of infection would be. Direct observations were made with an emphasis on the behavior of community members to understand how they might become infected. There are two possible explanations for this finding. First, these children may not recognize the danger of some adopted behaviors, such as dumping trash into watercourses. This may expose their legs or bodies to a stream of water. Secondly, the stigma associated with using watercourses or being infected with *S. mansoni* may have contributed to the children’s denial of contact with watercourses. The site of contact is supposed to be associated with acquiring infection, 23.6% of children who were contacting the center of the canal got schistosomiasis compared to 10.5% of those contacting the bank.

### Health facilities within the villages

The limited accessibility to diagnostic, chemotherapeutic, and preventive services significantly constrains the health-seeking behavior of people infected with schistosomiasis and other infectious diseases, particularly in developing countries. In addition to health illiteracy, the costs of travel and health service fees, geographic distance, social factors, and the frequent unavailability of services are among the most significant obstacles that individuals face when attempting to access health services [43, 44].

In this study, a small proportion (12%) of the population reported visiting the local health care unit in the villages, although it was accessible to the population within its catchment area. This issue was investigated while providing health education sessions. Stakeholders and fathers of screened children reported that doctors are not always available and the unit is severely under-resourced. Others stated that drugs are dispensed to relatives and acquaintances.

### Environmental factors

Our results shed light on the significant seasonal variation in the number of collected snails, including *B. alexandrina*, which is correlated with significant seasonal variations in temperature, salinity, turbidity, TC, and EC. Another important finding was that the type of vegetation had a significant effect on population density; duckweeds and grasses had a significant association with the presence of snails, which may be due to their importance as a food source, and snails may attach themselves to various plant parts to avoid the direct effect of sunlight, feed, or gain access to oxygen [45]. It is unknown to what extent snail population growth is attributable to these studied factors, as environmental changes measured across months were not always correlated with the total number of collected snails. However, this finding should shed light on other environmental factors implicated in snail survival. On equal terms, Monde et al., [46] reported that no single environmental parameter is a major determinant of host snail distribution, however, environmental parameters can account for 41 to 43% of the variation in snail density.

Due to the grant’s limited duration and budget, we were unable to cover all seasons and months. During the summer, schools were inaccessible due to summer vacation, and patients were recruited from mass gathering sites such as markets. Some children who were screened did not provide three consecutive daily stool samples; however, we collected three stool samples above the minimum required sample size to compensate for the dropouts. In addition, the shedding technique was adopted to diagnose snail infection, although this conventional method yields less diagnostic accuracy than other more advanced techniques, such as molecular techniques. Lastly, we were unable to provide PZQ to the infected children due to the MOHP’s treatment policy, which states that only the MOHP should administer treatment. As a result, we have provided the health authorities with a comprehensive report containing the names of infected children to treat them.

To the best of our knowledge, this is the first report to examine environmental factors implicated in the persistent transmission of schistosomiasis at late stages of elimination in hotspots in Egypt. Additionally, the study validated the concept of community engagement, as community members were interviewed to address the alleged barriers to the elimination of schistosomiasis, and their perspectives on causes of persistent transmission were revealed. Consequently, these factors were taken into account when constructing the questionnaire. The fact that the research team consisted of five different specialties affiliated with four institutes and organizations was an additional strength. This factor facilitated effective collaboration in highlighting the interaction between epidemiological, malacological, environmental factors, and human infection. Lastly, the research team provided citizens with health education sessions on how to safely interact with watercourses, how to avoid infection, how to detect disease symptoms early, and where to seek medical care.

## Conclusions

*S. mansoni* infection continues to circulate in many hotspot areas in Kafr El-Sheikh, Egypt. The prevalence of human infection is higher in the fall than in the summer. However, most cases are of light intensity. Persistent transmission is associated with sociodemographic factors such as young age (6 –10 years), male sex, and low maternal education level. In addition, residents continue to engage in risky behaviors, such as using water canals for laundry, land irrigation, water collection, bathing, and garbage disposal. A malacological survey revealed that the number of *B. alexandrina* varies across seasons, being higher in summer than in fall. Snail growth upsurges with an increase in water pH and decreases if water temperature or turbidity increases. The presence of grasses or duckweed is associated with increased snail infection. To sum up, schistosomiasis elimination in hot spot areas requires an integrated control approach that combines preventive chemotherapy with other complementary measures. Frequent MDA and up-to-date ecological studies are urgently required to assess the prevalence change and drug susceptibility in such areas.

## Data Availability

The datasets used and/or analyzed during the current study are available from the corresponding author on reasonable request.

## Abbreviations

HIPH: High Institute of Public Health
MDA: Mass drug administration
MOHP: Ministry of Health and Population
NTU: Nephelometric turbidity unity
PZQ: Praziquantel
TC: Total coliforms
TDS: Total dissolved solids
WHO: World Health Organization
